# The Evolving Role of Artificial Intelligence and Machine Learning in the Wearable Electrocardiogram: A Primer on Wearable-Enabled Prediction of Cardiac Dysfunction

**DOI:** 10.3390/bioengineering13020167

**Published:** 2026-01-29

**Authors:** Aditya Dave, Amartya Dave, Issam D. Moussa

**Affiliations:** 1 Carle Heart and Vascular Institute, University of Illinois Urbana-Champaign, Urbana, IL 61801, USA; imoussa@illinois.edu; 2 School of Medicine, University of California San Francisco, San Francisco, CA 94143, USA; amartya.dave@ucsf.edu

**Keywords:** Electrocardiography, artificial intelligence, machine learning, deep learning, wearable technology, signal processing, atrial fibrillation, ischemia, myocardial infarction, heart failure

## Abstract

The growing number of wearable electrocardiogram (ECG) users today, combined with the surge of artificial intelligence (AI) and machine learning (ML) in medical signal-processing, has led to a new age of wearable-enabled monitoring for cardiac conditions. With the development of advanced processing methods, wearables offer the opportunity to monitor and predict the probability of various cardiac conditions, from cardiac ischemia to arrhythmias, by collecting personalized data from the comfort of a user’s home. Although such technology has not yet entered the market, AI and ML research training specifically on wearable-based ECG data has grown significantly in the last decade. Despite this growing niche, there are few current articles reviewing the applications of these techniques in wearable ECG technology. To fill this gap, this article first primes the reader to the practical tools required to build models from ambulatory ECG, synthesizes the state of the field across major cardiac condition use-cases, and finally highlights recurring limitations in the current literature and outlines the need to improve reliability if this technology were to be widely utilized. As a result, we aim to help readers who otherwise may be unfamiliar with the specifics of these tools and their applications to form an interpretation of the current capabilities of AI/ML in wearable ECGs and identify key steps required for improvement based on the most current research.

## 1. Introduction

**Figure 1:**
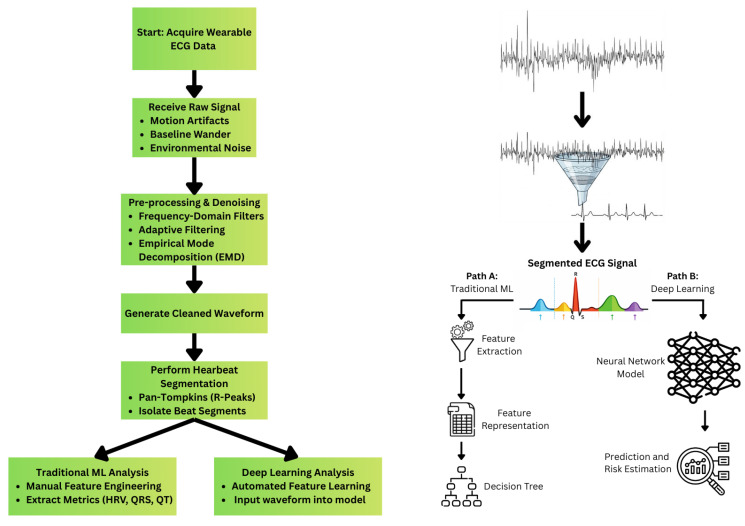
Summary of Typical ML-ECG Workflow.

Cardiovascular disease remains the leading cause of death around the world. Despite the prevalence of cardiovascular disease, medical advancements in prevention, diagnosis, and treatment allow patients to live longer, healthier lives than before. The development of wearable devices now allows individuals to continuously “monitor” their health and generate health-based insights based on their biodata. With the growth of continuous wearable monitoring, researchers have explored ways to utilize health signal data to better detect and favorably modulate the course of disease. Many wearables enable users to capture their own electrocardiogram (ECG) data, allowing researchers to explore how ambulatory ECG signals can be used to detect and ultimately provide early intervention for events such as arrhythmias, heart attacks, and even long-term cardiac ischemia. With the advent of artificial intelligence (AI) and machine learning (ML), it is now possible to construct highly accurate predictive models from wearable ECG data. Figure 1 summarizes a typical machine-learning workflow for ECG data.

Most existing reviews in this field emphasize either commercial device validation and regulatory clinical use [1], real-time monitoring and implementation systems [2], multimodal wearables rather than ECG specifically [3], or broad catalogs of AI/ML tasks and architectures [4]. Additionally, existing reviews often overlook the technical and physiological nuances of the single-lead ECG signal and lack a critical analysis of the translational gap between in silico performance and the reality of ambulatory application, often overinflating the present-day maturity of wearable AI-ECG systems. What is still needed, and what this review provides by identifying cross-cutting methodological failure modes, is a wearable-ECG–centered lens on the field’s clinical evolution, key technical constraints, and translational path forward, written for scientifically minded readers who may not be familiar with common signal-processing and machine-learning workflows. Accordingly, this review is structured as a primer plus translational synthesis focused on wearable ECG–based AI/ML for prediction and detection of clinically meaningful cardiac disease. We first orient the reader to the practical tools required to build models from ambulatory ECG (preprocessing, model families, and evaluation), and then synthesize the state of the field across major use-cases in this niche—spanning ischemia/MI, ventricular dysfunction and heart failure, and arrhythmia and malignant event risk.

### Key Objectives of This Review

- Provide a primer on wearable single-lead ECG characteristics and preprocessing/segmentation commonly used in AI/ML pipelines.
- Review AI/ML methods and the evaluation metrics used to assess performance, especially in imbalanced settings.
- Synthesize key wearable ECG clinical applications and conclude with the major limitations and translational considerations for real-world clinical integration.

## 2. Understanding Wearable ECG Data: Signal Processing, Techniques, Challenges

Electrocardiography (ECG) is considered the gold standard for non-invasive cardiac monitoring and is traditionally performed using a 12-lead system of electrodes [5]. Advances in sensor miniaturization and signal processing have enabled ECG acquisition in wearable technology, including smartwatches (Apple Watch, Samsung Galaxy Watch), fitness trackers (Fitbit, WHOOP), adhesive patch monitors, and smart textiles. These devices operate by using fewer leads than the traditional 12-lead system. Most wearables utilize single-lead systems, which offer advantages in portability and continuous monitoring, but drawbacks in resolution and sensitivity, most notably in spatial information. In the single-lead setup, often Lead I or Lead II, the general morphology of the P wave, QRS complex, and T wave is conserved, but minute details required for certain diagnostic criteria may be difficult to identify [6,7].

The quality of an ECG wearable signal depends on several parameters, including motion artifacts, baseline wander, and environmental factors. As the single-lead ECG is a relatively weak, continuous signal, noisy elements must be identified and filtered out before interpretation [8]. Out of such noise, motion artifacts, created by physical movement, are one of the most difficult to remove, as they usually overlap with the spectral components of the ECG signal itself [9]. Motion artifacts are also activity dependent; for example, obstacle course and circuit training will have different impacts on signal integrity compared to treadmill walking [10]. The type of activity also influences electrode-skin contact. Proper adhesion, stretchability, and conformability of the wearable electrode are crucial in maintaining high transmission quality or Signal-to-Noise Ratio (SNR) of electrophysiological signals [11].

Given these challenges, signal processing techniques are essential for converting noisy, raw signals into interpretable forms for further analysis. To address the inherent noise in wearable ECG data, three tools are employed: frequency-domain filtering (bandpass, highpass, lowpass), adaptive filtering, and Empirical Mode Decomposition (EMD) (Table 1).

A bandpass filter is a fundamental tool for removing noise by allowing a specific range of frequencies through. In ECG signals, a bandpass filter can be used to remove muscle artifacts and background noise like powerline interference. High-pass filters and low-pass filters can also be used to eliminate low-frequency noise caused by respiration or movement and remove high-frequency electrical interference, respectively. For real-time applications, adaptive filtering offers dynamic noise reduction [12,13,14]. For example, the Kalman filter, which uses previous outputs as current input, improves filtering over time [15]. Adaptive filtering allows the removal of noisy artifacts while preserving important diagnostic features of the ECG. Finally, EMD can further distinguish noise types by decomposing the signal into a set of components called intrinsic mode functions (IMFs) based on the signal’s own timeframe.

Once the signal is cleaned, the continuous waveform can be segmented into individual heartbeats. The Pan-Tompkins algorithm is a well-established method for segmentation that detects the R-peak and QRS complex based upon analyses of slope, amplitude, and width. This enables the extraction of clinically relevant features that are essential in ECG analysis and the performance of ML models. By applying a series of filters and implementing moving-window integration, the algorithm detects key cardiac events by identifying transient features [16].

Then, segmented beats are used in feature engineering, a manual process in which specific metrics are extracted from beats and fed into traditional ML models [17]. These features range from time-domain features such as QRS duration or QT interval to variability features like Heart Rate Variability (HRV). While segmentation and feature detection are important for traditional models, newer deep-learning architectures can learn directly from filtered ECG waveforms. By incorporating feature learning directly into classification, deep-learning models bypass explicit steps and capture subtle patterns that humans may miss—a major advancement in the field [18].

## 3. Artificial Intelligence and Machine Learning in Wearables

AI in ECG analysis initially relied on traditional feature-dependent ML and subsequently transitioned to unsupervised deep-learning (DL) methods. Decision-tree models, such as random forests and support vector machines (SVMs), and linear models like logistic regression, are the primary methods for classifying arrhythmias, particularly atrial fibrillation (AF). Such models rely on manual feature engineering for the cleaned ECG waveform and inputting those features into the models themselves, whereas unsupervised methods work independently. A 2024 study by Alimbayeva et al. found that among logical regression, decision trees, random trees, SVMs, and Convolutional Neural Networks, Convolutional Neural Networks demonstrated the highest accuracy when predicting cardiac diseases [19].

Although traditional methods are effective for specific diagnoses, the consensus is moving towards DL and AI as they emulate human cognitive processes with data recognition and therefore can handle diverse data and varying ECG diagnoses [20]. DL offers significant advantages by bypassing the need for manual feature detection. Neural networks, a type of advanced DL architecture, can extract features directly from raw ECG signals and learn from them.

The most common architectures are Convolutional Neural Networks (CNNs) and Recurrent Neural Networks (RNNs). A CNN is a neural network characterized by convolutional structures that allow it to perform well in the image processing field [21]. This type of model has been applied to various diseases, and its local connectivity and weight-sharing properties make it ideal for ECG analysis [20,22]. On the other hand, the RNN’s strengths lie in processing temporal and sequential data because of its feedback loops and memory of historical information. Oftentimes, in ECG data, they are utilized for rhythm analysis but require preprocessed features in the time and frequency domains [21]. These two architectures can also be used in tandem, creating efficient approaches to the classification of ECG data. In a study investigating DL of ECG for hypoglycemic events, researchers found that a CNN + RNN model performed better than a simple CNN system, with higher sensitivity and specificity [23]. A comparison of common algorithms can be seen in Table 2.

To ensure the reliability of these models and better understand their performance, they must be evaluated using a range of standardized metrics [24]. As model evaluation is essential for both research and commercial validation, many major studies and companies, such as Apple, AliveCor, and Fitbit, use the same metrics to assess performance.

Classification metrics can be organized into three main groups: binary, multiclass, and multilabel. Binary classification focuses on predicting if a sample belongs to one of two distinct groups. These values are commonly input into a 2 × 2 table known as a confusion matrix, which is then used to calculate a series of other metrics, like sensitivity (also known as recall), accuracy, and precision. Table 3 summarizes these classification metrics.

Another way to understand model performance is through a Receiver Operating Characteristic (ROC) curve, which illustrates the relationship between sensitivity and the false-positive rate. The area under the ROC curve (AUC-ROC) quantifies this performance, where 1 is perfect, and 0.5 is random chance [25]. These metrics have been summarized in Table 3.

Multiclass classification uses these principles but extends them to more than two categories, which involves macro-averaging (each class is equally weighted) and micro-averaging (weighted by the number of samples per class) to compute the same metrics, and the F1 score (which calculates a balanced measure of a model’s accuracy by taking the harmonic mean of precision and recall). Multilabel classification, however, is much more complicated, as a single ECG recording could have multiple diagnoses simultaneously. In such cases, metrics such as Hamming loss (fraction of misclassified instance-label pairs), subset accuracy (the multilabel counterpart of traditional accuracy), and one-error (the fraction of examples whose top-ranked label is not in the label set) are more suitable [26].

Beyond the standard accuracy-based measures, due to the skewed and imbalanced datasets in ECG analysis, alternate metrics are valuable. When class sizes differ largely, the previously mentioned F1 score and the Matthews Correlation Coefficient (MCC) offer a more balanced assessment [27]. Furthermore, precision-recall (PR) curves offer a way to understand the trade-off between positive predictive value and recall. Compared to ROC curves, PR curves focus on the performance of the positive class and thus are more informative in highly imbalanced datasets, where correctly identifying the minority class (e.g., diseased patients) is clinically more important than overall accuracy [25].

There are, however, limitations of wearable ECGs that limit their current adoption. Models that are trained on inherently noisy wearable data require thorough preprocessing and architectures capable of handling such imperfect samples. Secondly, the availability and quality of large, physician-annotated datasets are a major barrier to developing applications. Unlike atrial fibrillation, where vast, de-identified datasets are publicly available, processes like cardiac ischemia are far more challenging to create datasets for, forcing companies and researchers to invest significant time and money into labeling, organizing, and creating a new public dataset to tackle a different disease. Most public ECG databases like PhysioNet’s MIT-BIH Arrhythmia Database [28], PTB-XL [29], Chapman University Shaoxing [30], and the China Physiological Signal Challenge [31] are limited in size and diversity of disease, making it challenging to train models that generalize across patient populations and different disease states [32].

Another key challenge lies in the interpretability of models. Powerful deep-learning models often function as “black boxes” that make predictions without showing a clear step-by-step rationale. The lack of clear criteria under which black box models operate not only makes it difficult for clinicians to understand and adopt them but also makes regulatory approval challenging, especially in high-stakes fields such as cardiac health [33]. Addressing these challenges requires interdisciplinary work to ensure reliable, ethical, and actionable deployment into the healthcare space [34].

## 4. Applications in Detecting Cardiac Conditions: Arrhythmias, Heart Disease, and Others

### 4.1. Atrial Fibrillation (AF)

AF is a benchmark application of AI and ML in the wearable ECG realm. Over the last 10–15 years, AF has received intense focus among the scientific community due to the clear clinical need: AF is the most common arrhythmia worldwide, a major risk factor for stroke, and is often asymptomatic [35,36].

Large prospective studies were the first to confirm this approach at a population level. The Apple Heart Study, with nearly 420,000 participants, demonstrated how an algorithm deployed on a smartwatch and based on a photoplethysmography (PPG) sensor, which passively measures changes in blood flow, could identify possible AF and prompt physician follow-up. This study, verified using an ECG patch, served as a foundational proof-of-concept for an app-based wearable AI-screening system for cardiovascular disease preventative care [37].

ML–based wearables now demonstrate strong accuracy in detecting AF using single-lead ECGs and PPG signals [38,39]. Across major trials and meta-analyses, validated algorithms report sensitivities and specificities at or above 90% for distinguishing AF from a normal sinus rhythm (Table 4). Such high performance has been seen in large pragmatic studies (Perez et al., 2019, which uses an irregular rhythm classifier based on PPG data), population-level cohorts using wrist PPG devices (Guo et al., 2019), and randomized trials with handheld ECG screening (Halcox et al., 2017, AliveCor’s feature-based classifier using QRS interval analysis) [37,40,41]. Given such accuracy, some believe that deep-learning models have achieved cardiologist-level rhythm classification from single-lead ECGs, solidifying the capability and reliability of AI in arrhythmia detection [42]. However, translating these figures to broad screening remains challenging. While the REHEARSE-AF study (Halcox et al., 2017 [41]) demonstrated a four-fold increase in AF detection compared to routine care, the underlying automated algorithm was prone to false positives, leading to a reported real-world positive predictive value (PPV) as low as 5% for automated notifications before clinician overread. This disparity highlights the validation gap between FDA-cleared sensitivity (98%) and the practical burden of false-positive alerts in an unmonitored ambulatory population.

The field is now expanding beyond binary tasks of detection and is evolving into identifying nuance in disease progression for prognosis and risk stratification.

This shift is enabled by the continuous nature of wearable devices, allowing for the calculation of metrics like AF burden (the total amount of time a patient spends in AF). For instance, smartphone ECG burden measurement during the ‘blanking period’ post-ablation has shown that higher burden strongly predicts arrhythmia recurrence [43]. Emerging applications like these suggest that wearables hold potential to provide richer clinical insights past basic detection and into AF progression.

Moreover, AF research benefits from large open datasets such as the PhysioNet MIT-BIH Atrial Fibrillation Database, the AF Classification Challenge 2017 dataset, and the PhysioNet AF Prediction Database, which provide tens of thousands of annotated single-lead ECG segments for ML training and validation [28,44,45,46]. The availability of real-world data is far greater for AF than for many other arrhythmias, making AF especially well-suited for ML development.

Overall, AF represents the most validated and widely studied application of AI in wearable ECG monitoring. The lessons learned in AF detection have developed an ideal approach for tackling the more complex cardiac events and conditions that follow.

### 4.2. Myocardial Infarction

Myocardial Infarction (MI) affects roughly 15.9 million people worldwide every year [47]. Traditional diagnosis relies on a standard 12-lead ECG to identify characteristic changes such as ST elevation, limiting monitoring to clinical settings. Therefore, a major goal for wearables has been to replicate the diagnostic power of a 12-lead ECG in a single-lead device [48]. Early detection of MI, especially ST-elevated MI (STEMI), is critical for timely treatment, making a wearable solution using AI hold promise for pre-hospital triage. However, in comparison to arrhythmias, this challenge is significantly more complex.

Recent studies are demonstrating that this leap is possible. By comparing single-lead ECG deep-learning models to 12-lead models, studies are examining whether a single lead contains sufficient information for ML models to operate effectively. In an advanced single-lead study, Ezz explores the classification of MI and finds that single-lead deep-learning models like VGG16 and MobileNetV2 reach F1 scores of 97%, affirming that single-lead ECGs carry sufficient discriminatory power to classify MI [49].

This affirmation, however, comes with a trade-off between the convenience of a single lead and the comprehensive spatial information of the 12-lead ECG, and a significant performance gap still exists between laboratory benchmarking and ambulatory deployment [50]. This loss of information directly affects diagnostic accuracy, and studies on ST-segment monitoring have confirmed this. A study of patients with unstable coronary syndromes demonstrated that a single-lead ECG failed to detect 42% of events, highlighting how oftentimes more than a single lead is required to detect ongoing ischemia [51]. As such, there has been a need for a computational method to overcome the spatial context that is lost when using a single lead. One major avenue of research involves feature learning, where models attempt to identify markers invisible to the human eye [52].

A 2024 study by Anwar et al. developed an 11-class MI localization system and a lightweight, optimized autoencoder-k-NN classifier using only single-lead ECG features, achieving 99.7% accuracy and 99.2% F1-score [53]. Although the classifier reported nearly perfect metrics, it is important to note that it was trained on the curated, low-noise PTB-Diagnostic dataset. Similarly, a recent arXiv preprint proposes alignment techniques to situate single-lead ECG data in a multi-lead context to improve MI detection through single-lead machines. Experimentally, their model achieved “superior performance over baseline models across nine myocardial infarction types while maintaining a simpler architecture and lower computational overhead” [54]. Gibson and colleagues have supported this approach, with their group creating a model, using only lead II, yielding around 90.5% accuracy [55].

The other strategy lies in pure signal reconstruction, in which AI rebuilds a full 12-lead ECG from just a single lead [56]. In a recent 2025 article, Presacan and colleagues attempted to reconstruct a 12-lead electrocardiogram from limited leads (single and dual) but identified how models simply regressed to population averages rather than being sensitive to individual patient characteristics [57]. Although this study explored the mathematics of lead conversion using AI, it supports evidence of the fundamental void of data when using a single lead to pinpoint specific conditions.

Assisted by modern architectures and techniques, the MI domain has seen meaningful progress toward single-lead, real-time ECG detection of ischemia, with some models pushing performance past clinical accuracy. However, these in silico results often fail to account for the signal-to-noise ratio degradation inherent in wearable use, and a substantial gap remains in translating in silico models that are trained on clean, static datasets to inherently noisy ambulatory applications. When subjected to noisy, real-world settings, model performance often crashes. A 2024 AI model specifically designed to detect STEMI from noisy single-lead recordings achieved a 0.828 AUROC for detection but a very low specificity of 43.0% [58]. This suggests a high rate of false alarms, which raises concerns for a potential pre-hospital triage tool that must have high specificity. Furthermore, this quantitative disparity between lab and ambulatory applications underscores a fundamental limitation: while the electrical signal of an MI may be present in a single lead, it is frequently obscured by motion artifacts and baseline wander that curated datasets like PTB-XL do not replicate. Such models also require further validation in real-world trials where they can be verified with ground truth clinical outcomes to take the jump and integrate with the watches of the public. Therefore, future work in MI detection should focus on diverse datasets, validating wearable studies, and running outcome trials to ultimately translate these powerful innovations into clinical practice and improve patient outcomes.

### 4.3. Heart Failure

Compared to acute events, heart failure (HF) represents a greater detection challenge given the multi-factorial disease process and lack of HF-specific ECG changes. The need for specialized equipment to determine structural changes and ventricular function limits population-sized screenings. However, the fusion of single-lead wearable ECG data with AI offers a scalable and highly accessible screening solution for identifying at-risk HF patients [59].

A key application of wearable ECGs lies in detecting Left Ventricular Systolic Dysfunction (LVSD), a condition traditionally diagnosed via echocardiography and defined as LVEF ≤ 40%. Although single-lead ECGs do not measure structural properties of the heart, ML models can be trained to detect subtle electrical signals associated with reduced cardiac pump function (a structural and mechanical property). For example, Attia et al. (2022) [60] demonstrated how a CNN, initially trained on 12-lead and then adapted for a single-lead, can identify low ejection fractions, achieving an AUC of 0.885. When using the mean model predictions, the model achieved a sensitivity of 68.8% and a specificity of 83.7% at the optimal threshold of 0.67. Notably, at a lower cut-value of 0.60, sensitivity increased to 87.5% (specificity 80.7%), demonstrating the flexibility of the model as a high-sensitivity screening tool even in a cohort where 75% of patients (12 out of 16) had minimal or no symptoms of LVSD [60,61]. Using Apple Watch ECGs, this study validates how wearable data acquired in non-clinical settings holds utility in identifying patients with structural cardiac abnormalities. Models trained in a large cohort study by Sato and colleagues to identify left ventricular hypertrophy and low ejection fraction confirm this, achieving significantly higher accuracy and specificity than cardiologists interpreting the same Lead I data. The model showed a notable advantage in identifying low ejection fraction, with an accuracy of 78.3% (sensitivity 68.9% and specificity 93.3%), significantly outperforming Lead I cardiologists (65.6% accuracy, 53.7% sensitivity, 78.6% specificity) with *p* = 0.0127, *p* = 0.0379, and *p* = 0.0370 for accuracy, sensitivity, and specificity, respectively. This study quantitatively suggests that an automated model can interpret single-lead ECG data more reliably than a human expert, offering a reliable solution for identifying high-risk structural cardiac conditions in wearable settings [62].

ML, though, is extending beyond cross-sectional diagnosis and into long-term risk prediction [63]. The previously described AI-ECG CNN algorithm was found to be a prognostic marker, independent of the patient’s given LVEF score. This model identified patients at risk of Major Adverse Cardiovascular Events (MACE) and all-cause mortality over a two-year period from a single-lead ECG recording, underscoring how AI can extract complex information missed by traditional human interpretation [64].

This predictive capability is most actionable when applied to continuous monitoring, where AI can track deviations from a patient’s baseline to predict rapid deterioration. When patient data deviates from its baseline, ML can predict the need for HF-based hospitalization with a predictive accuracy comparable to implanted devices. In the LINK-HF multicenter study, 100 patients recently discharged after heart-failure hospitalization were continuously monitored for up to 90 days, during which 27 heart-failure hospitalizations occurred (≈27% event rate). Using a personalized machine-learning model applied to wearable multisensor data, the system predicted impending heart-failure hospitalization with 76–88% sensitivity at approximately 85% specificity by generating alerts a median of 6.5 days before admission. False alerts were assessed on a per-day basis outside predefined pre-event windows, explicitly modeling clinician alert burden rather than repeated detections of the same hospitalization. Although absolute alert rates per patient-year were not reported, this framework demonstrates that noninvasive longitudinal monitoring can provide clinically meaningful early warning within a time window that may allow for intervention [65].

It is established that the AI-driven single-lead ECG is a powerful, non-invasive platform that can advance the current state of disease prediction beyond simple rhythm monitoring. Still, it is important to remember that these deep-learning models often function as “black boxes”, hiding the features that drive predictions and limiting the integration of these tools in clinical decision making [66].

### 4.4. Ischemia

Myocardial Ischemia is the leading cause of mortality worldwide [67]. Causing cardiac dysfunction and often preceding arrhythmias, MI, or sudden death, ischemia manifests in subtle ways [68]. Detecting such sporadic changes in real-world ambulatory settings with AI and wearables is therefore a largely unsolved challenge, with current models performing at low sensitivities [69].

Recent studies, however, still highlight potential for bridging ML to single-lead ECGs for ischemic heart disease detection. In 2024, Marzoog et al. aimed to compare bicycle ergometry assessments with a single-lead ECG and pulse wave before and after the physical stress test. Using ML methodology, they reported a higher sensitivity (0.755) and specificity (0.516) compared to the bicycle ergometry test, with a sensitivity of 0.484 and a specificity of 0.531. The constrained sample set in this study limits generalizability, though, and models often fail to sustain this type of performance in increased heterogeneous populations [70].

A 2025 scoping review emphasizes this discrepancy. While many deep-learning architectures like ResNet often achieve clinical-grade performance on clean ECG datasets, with median sensitivity, precision, and specificity of 98.4%, 99.8%, and 99.1%, respectively, nearly all studies were retrospective and conducted on low-noise, non-wearable datasets. None of the studies collected ambulatory ECG recordings, which commonly have false alarms and contaminated signals. Consequently, these exceptionally high performance metrics likely reflect a combination of spectrum bias, limited disease heterogeneity, and potential label leakage rather than deployable real-world performance [71,72]. In some datasets, “ground truth” labels are themselves derived from automated algorithms or prior predictive models rather than definitive clinical endpoints, which can lead models to reproduce upstream labeling heuristics rather than detect true ischemia. Robust validation should require comparison against endpoint-confirmed labels (e.g., coronary angiography findings or adjudicated clinical events) rather than solely proxy annotations. Thus, translating ML to wearable scenarios necessitates significant improvements in filtering, validation, and study design [73].

In practice, these limitations translate into major barriers to clinical adoption of AI-enabled wearable ECG systems. High false-alert rates arising from motion artifact and variable electrode contact are intrinsic to single-lead wearable ECG acquisition and translate directly into clinician alert fatigue and unsustainable triage workload. Because these effects correlate with activity, placement, and device design, performance observed in curated datasets systematically overestimates real-world usability. Compounding this gap, ambulatory labels are often temporally imprecise, and alerts frequently lack clear downstream clinical actions, further destabilizing reliability at deployment. As a result, adoption of AI-enabled wearable ECG has been constrained not by retrospective accuracy but by failure to deliver reliable, low-burden, and actionable alerts within routine clinical workflows.

The difficulty of this task and the fragility of such optimistic metrics were captured in 2023 by Ekenberg and colleagues. By comparing a single-lead ECG with a 12-lead ECG to detect reversible ischemia during a stress test, they highlighted the limitations of the current system’s reliance on ST-segment analysis for ischemia detection. With diagnostic sensitivities of 8.3% for single-lead and 12.5% for 12-lead ECGs, the results suggest fundamental problems with traditional ST-segment analysis, regardless of the number of leads [69]. So, this failure is not due to a lack of spatial information in the ECG recording, but rather to the insufficient or unreliable nature of the deviation analysis for detecting subtle ischemic changes. Additionally, the order-of-magnitude drop from >98% in silico sensitivity to <9% clinical sensitivity suggests that high reported performance in the literature may be inflated by spectrum bias—where models are trained on distinct, late-stage disease cases—or label leakage, where the AI identifies features of the recording setup rather than the ischemic pathology itself. Therefore, while AI and ML can detect ischemia in a vacuum, translating this to wearables requires shifting away from static datasets and toward prospective validation in noisy, heterogeneous clinical environments that mirror wearable use cases.

### 4.5. Long QT Syndrome and Ventricular Arrhythmias

The early and accurate detection of Ventricular Arrhythmias (VAs) and diseases such as Long QT syndrome (LQTS) is crucial, particularly in settings where continuous 12-lead ECG is impractical or inaccessible. Currently, most efforts focus on developing models based on 12-lead ECGs to identify patients with LQTS; however, there is promise in adapting these approaches for single-lead ECG applications [74,75,76].

As with other applications, the basic measurement of the QT interval and ST-T morphology must be validated on wearable ECG before being applied to AI or ML models. In a multicenter trial comparing Apple Watch and KardiaMobile 6L recordings with a reference 12-lead ECG in 98 patients with confirmed LQTS, researchers found a strong QTc (corrected QT interval) correlation (r = 0.7 for Apple Watch) and moderate agreement in ST-T morphology (k = 0.651 for KardiaMobile) [77]. Although performance was not indicative of replacing the 12-lead ECG, it elucidates potential in monitoring patients at home.

With measurements established, research is expanding into advanced tasks. In a recent analysis, a neural-network classifier trained on single-lead data discriminated major LQTS genotypes with high fidelity, further suggesting that single-lead device signals can capture meaningful information, uninterpretable to humans, like genotype-specific electrophysiologic signatures [78].

Another focus in the field is predicting potentially catastrophic events instead of recognizing an existing condition. In a retrospective study, Fiorina et al. illustrate how deep learning could predict near-term risk of sustained VAs from a single lead ambulatory device. Their model accurately predicted future ventricular tachycardia (VT) from recordings of sustained VT (≥180 b.p.m.) along with 90% of VT that degenerated into ventricular fibrillation. Paired with other advances in ventricular dysfunction detection, this model lays the groundwork for new approaches to combat sudden cardiac death (SCD) while improving patient outcomes by predicting potential cardiac events.

Still, hurdles exist for clinical integration. As single-lead systems only show moderate alignment with 12-lead systems, deep learning may amplify artifacts or noise in real-world settings. And, as with all AI-identified features, a black-box barrier to clinical entry lies ahead [79]. In addition, false detections in ambulatory monitoring, often driven by motion artifact, variable electrode contact, and context shifts (e.g., sleep, exercise, or posture), can create unacceptable triage workload and impede adoption through clinician alert fatigue if not explicitly managed. Accordingly, false detections should be managed with explicit supervision embedded in the system around the model. First, signal-quality gating can suppress predictions during poor contact or high-motion segments; second, temporal persistence rules (e.g., requiring sustained) to filter transient noise; third, context-aware checks (checking activity state and concurrent rhythm classification) to reduce spurious triggers; and fourth, human triage, where only high-confidence alerts can prompt clinician review while low-confidence signals trigger re-acquisition or monitoring. Prospective studies should therefore report workload-centered metrics (false alerts per patient-time, time-to-triage, downstream testing) alongside sensitivity and specificity and evaluate performance across heterogeneous devices and real-world contexts.

## 5. Conclusions

The evolution of artificial intelligence in single-lead wearable analysis has established it as a potentially valuable tool in cardiovascular medicine [80]. It is essential to recognize that the accuracy of an AI-ECG model is only as good as the data it has been trained on. Similarly, datasets are only as reliable as they are realistic to the real world (in which they often lack diversity) and the signal quality captured from a patient (which is normally contaminated with noise and artifacts). This features the reliance of such models on robust signal processing methods and ground-truth data sources, regardless of the complexity of the ML being used. To bridge the existing “translational gap,” future research must pivot from achieving high-accuracy benchmarks on static, curated datasets toward prospective validation in heterogeneous environments that more closely mirror “in-the-wild” deployment. This transition requires a rigorous commitment to reporting clinical metrics such as PPV and false-alarm rates alongside traditional AUC scores to provide clinicians with a realistic assessment of diagnostic precision in low-prevalence screening contexts. Furthermore, overcoming technical hurdles like spectrum bias and the “black-box” nature of deep learning will be essential for these tools to move from automated discovery to reliable, evidence-based clinical instruments. Despite this, the field of AI-enabled wearable technology for health has progressed far beyond its initial successes in simple rhythm detection. As commercial watches continue to become better integrated into the lives of millions worldwide, there is an urgent need for clinicians and the health system overall to grasp both the applications and limitations of such powerful technology.

This review first outlines the basics of wearable ECG data, providing an overview of signal processing (Table 1), key AI architectures, and how to evaluate them using classification metrics (Table 2). Then, we discussed the progress and challenges of AI applications: starting with the benchmark success of atrial fibrillation, trying to replicate a 12-lead ECG’s power for myocardial infarction detection, correlating the underlying electrical patterns to a mechanical disease in heart failure, and exploring the complex challenge of predicting lethal events like sudden cardiac death. Here, we demonstrate AI’s potential to move past replicating human-centric clinical markers and toward automated discovery of novel, oftentimes imperceptible biomarkers. However, as this review has covered, this potential is tied to fundamental challenges of data. There is an unbridged gap between high-performance in silico models and the realities of ambulatory applications.

The future of the field holds potential, but it currently stands at a juncture. The majority of high-performance metrics cited in the literature are derived from curated, retrospective datasets that do not account for the noise and signal variability of real-world ambulatory use. The translation from a powerful research strategy into a reliable clinical instrument for diagnosis and treatment will depend entirely on the next step of validation in prospective, large-scale, real-world trials.

## Figures and Tables

**Table 1:** Comparison of Key ECG Pre-processing and Segmentation Techniques.

Technique	Purpose and Pros	Limitations	Applications
Frequency-Domain Filters (Bandpass, Low-pass, High-pass)	Purpose: Remove noise at specific, known frequencies. Pros: Simple, computationally fast, and very effective for predictable noise.	Inflexible. Removes ECG information if it shares frequency with noise. Poor with unpredictable noise (motion).	Powerline interference, baseline wander, and high-frequency electrical noise.
Adaptive Filtering (e.g., Kalman Filter)	Purpose: Dynamically removes noise by adapting to the signal in real time. Pros: Effective at removing noise while preserving ECG features.	Computationally complex and resource-intensive.	Real-time motion artifacts.
Empirical Mode Decomposition (EMD)	Purpose: Decompose the signal into its base components (IMFs). Pros: Data-driven (uses the signal’s own properties). Separates noise sources.	Cons: Computationally intensive and complex implementation. Sensitive to noise.	Isolate and remove non-stationary noise (like motion) before analysis.
Pan-Tompkins Algorithm	Purpose: To segment the continuous signal into individual heartbeats. Pros: Well-established, reliable, and efficient for detecting QRS complexes.	Cons: Less effective for subtle P or T wave detection	Foundational step for feature engineering: calculating HRV or QRS duration.

**Table 2:** Comparison of Machine-Learning Architectures for ECG Analysis.

Model Type	Core Principle	ECG Analysis Pros	Limitations	Example Application
Traditional ML (e.g., SVM, Random Forest)	Manual feature engineering, then classification.	Specific, well-defined diagnoses.	Dependent on manually extracted features.	Arrhythmias, such as Atrial Fibrillation (AF).
Convolutional Neural Network (CNN)	Learning patterns directly from the (raw or processed) signal.	Ideal due to local connectivity and weight-sharing.	Difficult to interpret decision making	Wide range of cardiac diseases. Analyzing ECG as a 1D image.
Recurrent Neural Network (RNN)	Processes temporal and sequential data using “feedback loops and memory.”	Analyzing time-series data where the order of events matters.	Requires preprocessed features.	Rhythm analysis.
Hybrid Model (e.g., CNN + RNN)	Combines spatial strengths of CNNs with the sequential memory of RNNs.	Captures both types (time and spatial) of information.	Increased model complexity.	Detecting hypoglycemic events—showed higher sensitivity and specificity than a simple CNN.

**Table 3:** Classification Metrics for Evaluating ML Models.

Metric	What It Measures	Use Case	Key Limitation
Accuracy	The proportion of all predictions that were correct.	Quick assessment of balanced datasets.	Misleading on imbalanced datasets.
Sensitivity	The proportion of positive cases predicted as positive.	Screening. When there is a high cost of a false negative.	Ignores false positives.
Specificity	The proportion of negative cases predicted as negative.	Confirmatory tests. When there is a high cost of a false positive.	Ignores false negatives.
Precision	The proportion of true positives from all predicted positives.	Evaluating “noise” or the cost of a model’s alarms.	High on overly conservative models.
F1-Score	The harmonic mean of precision and recall.	Imbalanced datasets. Provides one balanced score between Sensitivity and Precision.	Less intuitive to explain than simple accuracy.
AUC-ROC	Measures trade-off between Sensitivity and the False-Positive Rate.	Comparing the general performance of models.	“Less informative in highly imbalanced datasets” than a PR curve.
PR Curve	The Precision-Recall (PR) Curve.	Imbalanced datasets where the positive class is the most important.	Not as commonly reported as AUC-ROC, but often more valuable for disease detection.
Confusion Matrix	Matrix of 4 outcomes: True Positives, False Positives, True Negatives, False Negatives.	Source for calculating other metrics.	Detailed breakdown of performance.

**Table 4:** Performance of classification algorithms integrated with wearable devices in detecting AF.

Study	Device	Classification System	Population	Performance
Perez et al., 2019 [37], ***NEJM***	Apple Watch	Tachogram Sampling and Irregularity Analysis (proprietary)	419,297 participants	PPV = 84%
Guo et al., 2019 [40], ***JACC***	Huawei smartwatch or wristband	PPG algorithm on pulse waves (proprietary)	187,912 users	PPV = 91.6%
Halcox et al., 2017 [41], ***Circulation***	AliveCor KardiaMobile	ECG algorithm (proprietary)	1001 older-adults	98% Sensitivity 97% Specificity 5% PPV (Real-world alarms)
Hannun et al., 2019 [42], ***Nat Med***	Zio monitor	34-layer DNN for ECGs	53,549 patients	F1 = 0.837 AUC = 0.97

## Data Availability

No new data were created or analyzed in this study. Data sharing is not applicable to this article.

## Abbreviations

The following abbreviations are used in this manuscript:

AF	Atrial Fibrillation
AI	Artificial Intelligence
AUC/AUC-ROC	Area Under the (ROC) Curve
CNN	Convolutional Neural Network
DL	Deep Learning
DNN	Deep Neural Network
ECG	Electrocardiogram
EMD	Empirical Mode Decomposition
HF	Heart Failure
HRV	Heart Rate Variability
IMFs	Intrinsic Mode Functions
LVEF	Left Ventricular Ejection Fraction
LVSD	Left Ventricular Systolic Dysfunction
LQTS	Long QT Syndrome
MACE	Major Adverse Cardiovascular Events
MCC	Matthews Correlation Coefficient
MI	Myocardial Infarction
ML	Machine Learning
PPV	Positive Predictive Value
PPG	Photoplethysmography
PR Curve	Precision-Recall Curve
RNN	Recurrent Neural Network
ROC	Receiver Operating Characteristic
SCD	Sudden Cardiac Death
SNR	Signal-to-Noise Ratio
STEMI	ST-Elevated Myocardial Infarction
SVM	Support Vector Machine
VA	Ventricular Arrhythmia
VT	Ventricular Tachycardia

## References

[1] Bouzid Z., Al-Zaiti S.S., Bond R., Sejdic E. (2022). Remote and Wearable ECG Devices with Diagnostic Abilities in Adults: A State-of-the-Science Scoping Review. Heart Rhythm..

[2] Gaoudam N., Sakhamudi S.K., Kamal B., Addla N., Reddy E.P., Ambala M., Lavanya K., Palaparthi E.C., Bhattam A., Periasamy P. (2025). Wearable Devices and AI-Driven Remote Monitoring in Cardiovascular Medicine: A Narrative Review. Cureus.

[3] Abedi A., Verma A., Jain D., Kaetheeswaran J., Chui C., Lankarany M., Khan S.S. (2025). AI-Driven Real-Time Monitoring of Cardiovascular Conditions with Wearable Devices: Scoping Review. JMIR Mhealth Uhealth.

[4] Neri L., Oberdier M.T., van Abeelen K.C.J., Menghini L., Tumarkin E., Tripathi H., Jaipalli S., Orro A., Paolocci N., Gallelli I. (2023). Electrocardiogram Monitoring Wearable Devices and Artificial-Intelligence-Enabled Diagnostic Capabilities: A Review. Sensors.

[5] Sattar Y., Chhabra L. (2025). Electrocardiogram. StatPearls.

[6] Bing P., Liu W., Zhai Z., Li J., Guo Z., Xiang Y., He B., Zhu L. (2024). A Novel Approach for Denoising Electrocardiogram Signals to Detect Cardiovascular Diseases Using an Efficient Hybrid Scheme. Front. Cardiovasc. Med..

[7] Witvliet M.P., Karregat E.P.M., Himmelreich J.C.L., de Jong J.S.S.G., Lucassen W.A.M., Harskamp R.E. (2021). Usefulness, Pitfalls and Interpretation of Handheld Single-lead Electrocardiograms. J. Electrocardiol..

[8] Satija U., Ramkumar B., Manikandan M.S. (2018). Automated ECG Noise Detection and Classification System for Unsupervised Healthcare Monitoring. IEEE J. Biomed. Health Inform..

[9] An X., Stylios G.K. (2020). Comparison of Motion Artefact Reduction Methods and the Implementation of Adaptive Motion Artefact Reduction in Wearable Electrocardiogram Monitoring. Sensors.

[10] Lindsey B., Snyder S., Zhou Y., Shim J.K., Hahn J.-O., Evans W., Martin J. (2025). Activity Type Effects Signal Quality in Electrocardiogram Devices. Sensors.

[11] Lim K., Seo H., Chung W.G., Song H., Oh M., Ryu S.Y., Kim Y., Park J.-U. (2024). Material and Structural Considerations for High-Performance Electrodes for Wearable Skin Devices. Commun. Mater..

[12] Fuadah Y.N., Lim K.M. (2025). Advances in Cardiovascular Signal Analysis with Future Directions: A Review of Machine Learning and Deep Learning Models for Cardiovascular Disease Classification Based on ECG, PCG, and PPG Signals. Biomed. Eng. Lett..

[13] Thakor N.V., Zhu Y.S. (1991). Applications of Adaptive Filtering to ECG Analysis: Noise Cancellation and Arrhythmia Detection. IEEE Trans. Biomed. Eng..

[14] Poungponsri S., Yu X.-H. (2013). An Adaptive Filtering Approach for Electrocardiogram (ECG) Signal Noise Reduction Using Neural Networks. Neurocomputing.

[15] Manju B.R., Sneha M.R. (2020). ECG Denoising Using Wiener Filter and Kalman Filter. Procedia Comput. Sci..

[16] Pan J., Tompkins W.J. (1985). A Real-Time QRS Detection Algorithm. IEEE Trans. Biomed. Eng..

[17] Singh A.K., Krishnan S. (2023). ECG Signal Feature Extraction Trends in Methods and Applications. Biomed. Eng. Online.

[18] Jahangir R., Islam M.N., Islam M.S., Islam M.M. (2025). ECG-Based Heart Arrhythmia Classification Using Feature Engineering and a Hybrid Stacked Machine Learning. BMC Cardiovasc. Disord..

[19] Alimbayeva Z., Alimbayev C., Ozhikenov K., Bayanbay N., Ozhikenova A. (2024). Wearable ECG Device and Machine Learning for Heart Monitoring. Sensors.

[20] Wu Z., Guo C. (2025). Deep Learning and Electrocardiography: Systematic Review of Current Techniques in Cardiovascular Disease Diagnosis and Management. Biomed. Eng. OnLine.

[21] Sun J. (2023). Automatic Cardiac Arrhythmias Classification Using CNN and Attention-based RNN Network. Heal. Technol. Lett..

[22] Goto S., Goto S. (2019). Application of Neural Networks to 12-Lead Electrocardiography—Current Status and Future Directions. Circ. Rep..

[23] Porumb M., Stranges S., Pescapè A., Pecchia L. (2020). Precision Medicine and Artificial Intelligence: A Pilot Study on Deep Learning for Hypoglycemic Events Detection Based on ECG. Sci. Rep..

[24] Rainio O., Teuho J., Klén R. (2024). Evaluation Metrics and Statistical Tests for Machine Learning. Sci. Rep..

[25] Erickson B.J., Kitamura F. (2021). Magician’s Corner: 9. Performance Metrics for Machine Learning Models. Radiol. Artif. Intell..

[26] Zhang M.-L., Zhou Z.-H. (2014). A Review on Multi-Label Learning Algorithms. IEEE Trans. Knowl. Data Eng..

[27] Chicco D., Jurman G. (2020). The Advantages of the Matthews Correlation Coefficient (MCC) over F1 Score and Accuracy in Binary Classification Evaluation. BMC Genom..

[28] Moody G.B., Mark R.G. (2001). The Impact of the MIT-BIH Arrhythmia Database. IEEE Eng. Med. Biol. Mag..

[29] Wagner P., Strodthoff N., Bousseljot R.-D., Kreiseler D., Lunze F.I., Samek W., Schaeffter T. (2020). PTB-XL, a Large Publicly Available Electrocardiography Dataset. Sci. Data.

[30] Zheng J., Chu H., Struppa D., Zhang J., Yacoub S.M., El-Askary H., Chang A., Ehwerhemuepha L., Abudayyeh I., Barrett A. (2020). Optimal Multi-Stage Arrhythmia Classification Approach. Sci. Rep..

[31] Wang X., Ma C., Zhang X., Gao H., Clifford G., Liu C. (2021). Paroxysmal Atrial Fibrillation Events Detection from Dynamic ECG Recordings: The 4th China Physiological Signal Challenge 2021. Proc. PhysioNet.

[32] Chung C.T., Lee S., King E., Liu T., Armoundas A.A., Bazoukis G., Tse G. (2022). Clinical Significance, Challenges and Limitations in Using Artificial Intelligence for Electrocardiography-Based Diagnosis. Int. J. Arrhythmia.

[33] Raposo V.L. (2025). The Fifty Shades of Black: About Black Box AI and Explainability in Healthcare. Med. Law. Rev..

[34] McCoy L.G., Brenna C.T.A., Chen S.S., Vold K., Das S. (2022). Believing in Black Boxes: Machine Learning for Healthcare Does Not Need Explainability to Be Evidence-Based. J. Clin. Epidemiol..

[35] Suomalainen O.P., Martinez-Majander N., Broman J., Mannismäki L., Aro A., Curtze S., Pakarinen S., Lehto M., Putaala J. (2023). Stroke in Patients with Atrial Fibrillation: Epidemiology, Screening, and Prognosis. J. Clin. Med..

[36] Chousou P.A., Chattopadhyay R., Tsampasian V., Vassiliou V.S., Pugh P.J. (2023). Electrocardiographic Predictors of Atrial Fibrillation. Med. Sci..

[37] Perez M.V., Mahaffey K.W., Hedlin H., Rumsfeld J.S., Garcia A., Ferris T., Balasubramanian V., Russo A.M., Rajmane A., Cheung L. (2019). Large-Scale Assessment of a Smartwatch to Identify Atrial Fibrillation. N. Engl. J. Med..

[38] Francisco A., Pascoal C., Lamborne P., Morais H., Gonçalves M. (2025). Wearables and Atrial Fibrillation: Advances in Detection, Clinical Impact, Ethical Concerns, and Future Perspectives. Cureus.

[39] Barrera N., Solorzano M., Jimenez Y., Kushnir Y., Gallegos-Koyner F., Dagostin de Carvalho G. (2025). Accuracy of Smartwatches in the Detection of Atrial Fibrillation. JACC: Adv..

[40] Guo Y., Wang H., Zhang H., Liu T., Liang Z., Xia Y., Yan L., Xing Y., Shi H., Li S. (2019). Mobile Photoplethysmographic Technology to Detect Atrial Fibrillation. J. Am. Coll. Cardiol..

[41] Halcox J.P.J., Wareham K., Cardew A., Gilmore M., Barry J.P., Phillips C., Gravenor M.B. (2017). Assessment of Remote Heart Rhythm Sampling Using the AliveCor Heart Monitor to Screen for Atrial Fibrillation. Circulation.

[42] Hannun A.Y., Rajpurkar P., Haghpanahi M., Tison G.H., Bourn C., Turakhia M.P., Ng A.Y. (2019). Cardiologist-Level Arrhythmia Detection and Classification in Ambulatory Electrocardiograms Using a Deep Neural Network. Nat. Med..

[43] Noujaim C., Lim C., Donnellan E., Mekhael M., Zhao C., Shan B., Hadi El Hajjar A., Chouman N., Assaf A., Feng H. (2023). Smartphone AF Burden During the Blanking Period Predicts Catheter Ablation Outcomes: Insights from DECAAF II. JACC Clin. Electrophysiol..

[44] Clifford G.D., Liu C., Moody B., Lehman L.-W.H., Silva I., Li Q., Johnson A.E., Mark R.G. AF Classification from a Short Single Lead ECG Recording: The PhysioNet/Computing in Cardiology Challenge 2017. Proceedings of the 2017 Computing in Cardiology (CinC).

[45] Moody G., Goldberger A., McClennen S., Swiryn S.P. Predicting the Onset of Paroxysmal Atrial Fibrillation: The Computers in Cardiology Challenge 2001. Proceedings of the Computers in Cardiology 2001.

[46] Goldberger A.L., Amaral L.A., Glass L., Hausdorff J.M., Ivanov P.C., Mark R.G., Mietus J.E., Moody G.B., Peng C.K., Stanley H.E. (2000). PhysioBank, PhysioToolkit, and PhysioNet: Components of a New Research Resource for Complex Physiologic Signals. Circulation.

[47] Ojha N., Dhamoon A.S. (2025). Myocardial Infarction. StatPearls.

[48] Obianom E.N., Ng G.A., Li X. (2025). Reconstruction of 12-Lead ECG: A Review of Algorithms. Front. Physiol..

[49] Ezz M. (2025). Deep Learning-Driven Single-Lead ECG Classification: A Rapid Approach for Comprehensive Cardiac Diagnostics. Diagnostics.

[50] Janciuleviciute K., Sokas D., Bacevicius J., Sornmo L., Petrenas A. (2026). ECG-Based Detection of Acute Myocardial Infarction Using a Wrist-Worn Device. IEEE Trans. Biomed. Eng..

[51] Drew B.J., Pelter M.M., Adams M.G., Wung S.F., Chou T.M., Wolfe C.L. (1998). 12-Lead ST-Segment Monitoring vs Single-Lead Maximum ST-Segment Monitoring for Detecting Ongoing Ischemia in Patients with Unstable Coronary Syndromes. Am. J. Crit. Care.

[52] Davarmanesh P., Lin Q., Tenison I., Jabbour G., Alam R. Detection of Acute Myocardial Infarction Using Deep Learning on Lead-I ECG Data. Proceedings of the 2024 IEEE 20th International Conference on Body Sensor Networks (BSN).

[53] Anwar S.M.S., Pal D., Mukhopadhyay S., Gupta R. (2024). A Lightweight Method of Myocardial Infarction Detection and Localization from Single Lead ECG Features Using Machine Learning Approach. IEEE Sens. Lett..

[54] Jin J., Fang X., Wang H., Li J., Liu C., Xie D., Li H., Hong S. (2025). Self-Alignment Learning to Improve Myocardial Infarction Detection from Single-Lead ECG. arXiv.

[55] Gibson C.M., Mehta S., Ceschim M.R.S., Frauenfelder A., Vieira D., Botelho R., Fernandez F., Villagran C., Niklitschek S., Matheus C.I. (2022). Evolution of Single-Lead ECG for STEMI Detection Using a Deep Learning Approach. Int. J. Cardiol..

[56] Savostin A., Koshekov K., Ritter Y., Savostina G., Ritter D. (2024). 12-Lead ECG Reconstruction Based on Data from the First Limb Lead. Cardiovasc. Eng. Technol..

[57] Presacan O., Dorobanţiu A., Isaksen J.L., Willi T., Graff C., Riegler M.A., Sridhar A.R., Kanters J.K., Thambawita V. (2025). Evaluating the Feasibility of 12-Lead Electrocardiogram Reconstruction from Limited Leads Using Deep Learning. Commun. Med..

[58] Dhingra L., Khunte A., Shankar S., Khera R. (2024). Abstract 4145357: Detecting ST Elevation Myocardial Infarction on a Noisy Single Limb-Lead ECG: An Artificial Intelligence-Enabled Approach Adaptable to Portable Devices. Circulation.

[59] Hasumi E., Fujiu K., Chen Y., Miyamoto S., Oida M., Shimizu Y., Kani K., Goto K., Uchida R., Liu Y. (2025). Heart Failure Monitoring with a Single-lead Electrocardiogram at Home. Int. J. Cardiol..

[60] Attia Z.I., Harmon D.M., Dugan J., Manka L., Lopez-Jimenez F., Lerman A., Siontis K.C., Noseworthy P.A., Yao X., Klavetter E.W. (2022). Prospective Evaluation of Smartwatch-Enabled Detection of Left Ventricular Dysfunction. Nat. Med..

[61] Attia Z.I., Kapa S., Lopez-Jimenez F., McKie P.M., Ladewig D.J., Satam G., Pellikka P.A., Enriquez-Sarano M., Noseworthy P.A., Munger T.M. (2019). Screening for Cardiac Contractile Dysfunction Using an Artificial Intelligence-Enabled Electrocardiogram. Nat. Med..

[62] Sato M., Kodera S., Setoguchi N., Tanabe K., Kushida S., Kanda J., Saji M., Nanasato M., Maki H., Fujita H. (2024). Deep Learning Models for Predicting Left Heart Abnormalities from Single-Lead Electrocardiogram for the Development of Wearable Devices. Circ. J..

[63] Dhingra L.S., Aminorroaya A., Pedroso A.F., Khunte A., Sangha V., McIntyre D., Chow C.K., Asselbergs F.W., Brant L.C.C., Barreto S.M. (2025). Artificial Intelligence-Enabled Prediction of Heart Failure Risk from Single-Lead Electrocardiograms. JAMA Cardiol..

[64] Alrumayh A., Bächtiger P., Sau A., Mansell J., Almonte M.T., Chhatwal K., Ng F.S., Kelshiker M.A., Peters N.S. (2025). Artificial Intelligence Analysis of the Single-Lead ECG Predicts Long-Term Clinical Outcomes. Eur. Heart J. Digit. Health.

[65] Stehlik J., Schmalfuss C., Bozkurt B., Nativi-Nicolau J., Wohlfahrt P., Wegerich S., Rose K., Ray R., Schofield R., Deswal A. (2020). Continuous Wearable Monitoring Analytics Predict Heart Failure Hospitalization. Circ. Heart Fail..

[66] Ayano Y.M., Schwenker F., Dufera B.D., Debelee T.G. (2022). Interpretable Machine Learning Techniques in ECG-Based Heart Disease Classification: A Systematic Review. Diagnostics.

[67] An X., Zhou N., Xie J., Liu C., Zhang F., Ouyang W., Wang S., Liu Z., Pan X. (2025). Global, Regional, and National Time Trends in Mortality for Ischemic Heart Disease, 1990–2019: An Age-Period-Cohort Analysis for the Global Burden of Disease 2019 Study. Rev. Cardiovasc. Med..

[68] Shimokawa H., Yasuda S. (2008). Myocardial Ischemia: Current Concepts and Future Perspectives. J. Cardiol..

[69] Fabricius Ekenberg L., Høfsten D.E., Rasmussen S.M., Mølgaard J., Hasbak P., Sørensen H.B.D., Meyhoff C.S., Aasvang E.K. (2023). Wireless Single-Lead versus Standard 12-Lead ECG, for ST-Segment Deviation during Adenosine Cardiac Stress Scintigraphy. Sensors.

[70] Marzoog B.A., Chomakhidze P., Gognieva D., Silantyev A., Suvorov A., Abdullaev M., Mozzhukhina N., Filippova D.A., Kostin S.V., Kolpashnikova M. (2025). Development and Validation of a Machine Learning Model for Diagnosis of Ischemic Heart Disease Using Single-Lead Electrocardiogram Parameters. World J. Cardiol..

[71] Davis S.E., Matheny M.E., Balu S., Sendak M.P. (2023). A Framework for Understanding Label Leakage in Machine Learning for Health Care. J. Am. Med. Inf. Assoc..

[72] Tseng A.S., Shelly-Cohen M., Attia I.Z., Noseworthy P.A., Friedman P.A., Oh J.K., Lopez-Jimenez F. (2021). Spectrum Bias in Algorithms Derived by Artificial Intelligence: A Case Study in Detecting Aortic Stenosis Using Electrocardiograms. Eur. Heart J. Digit. Health.

[73] Kim A., Chatterjee M., Iansavitchene A., Komeili M., Chan A.D.C., Yang H., Chui J. (2025). Artificial Intelligence for Electrocardiographic Diagnosis of Perioperative Myocardial Ischaemia: A Scoping Review. Br. J. Anaesth..

[74] Bos J.M., Attia Z.I., Albert D.E., Noseworthy P.A., Friedman P.A., Ackerman M.J. (2021). Use of Artificial Intelligence and Deep Neural Networks in Evaluation of Patients with Electrocardiographically Concealed Long QT Syndrome from the Surface 12-Lead Electrocardiogram. JAMA Cardiol..

[75] Jiang R., Cheung C.C., Garcia-Montero M., Davies B., Cao J., Redfearn D., Laksman Z.M., Grondin S., Atallah J., Escudero C.A. (2024). Deep Learning-Augmented ECG Analysis for Screening and Genotype Prediction of Congenital Long QT Syndrome. JAMA Cardiol..

[76] Alam R., Aguirre A., Stultz C.M. (2024). Detecting QT Prolongation from a Single-Lead ECG with Deep Learning. PLoS Digit. Health.

[77] Delinière A., Bessière F., Placide L., Pasquié J.-L., Haddad C., Tirel S., Mokhtar H., Morel E., Gardey K., Dulac A. (2024). Wearable Electrocardiogram Devices in Patients with Congenital Long QT Syndrome: The SMART-QT Study. Arch. Cardiovasc. Dis..

[78] Srutova M., Kremen V., Lhotska L. (2025). Electrocardiographic Discrimination of Long QT Syndrome Genotypes: A Comparative Analysis and Machine Learning Approach. Sensors.

[79] Kwon J., Jo Y.-Y., Lee S.Y., Kim K.-H. (2021). Artificial Intelligence Using Electrocardiography: Strengths and Pitfalls. Eur. Heart J..

[80] Smith S., Maisrikrod S. (2025). Wearable Electrocardiogram Technology: Help or Hindrance to the Modern Doctor?. JMIR Cardio.

